# Depression and attempted suicide under pregabalin therapy

**DOI:** 10.1186/s12991-014-0037-8

**Published:** 2014-12-04

**Authors:** Andreas Kustermann, Cornelia Möbius, Timo Oberstein, Helge H Müller, Johannes Kornhuber

**Affiliations:** Department of Psychiatry and Psychotherapy, Friedrich-Alexander University of Erlangen-Nuremberg, Schwabachanlage 6, 91054 Erlangen, Germany; Department of Neurology, Friedrich-Alexander University of Erlangen-Nuremberg, Schwabachanlage 6, 91054 Erlangen, Germany

**Keywords:** Depression, Anticonvulsants, Suicide, Pregabalin

## Abstract

Originally developed for the treatment of epilepsy, pregabalin has become a compound with a wide spectrum of indications comprising anxiety disorders and chronic pain and is therefore largely prescribed. Thus, it is important for clinicians to be aware of rare, but serious adverse effects. The following report illustrates the case of a 20-year-old male with a severe depressive syndrome following pregabalin medication which even led to a suicide attempt.

## Background

Pregabalin is a compound originally developed for treating epilepsy. Meanwhile, it has shown positive effects on neuropathic pain as well as on general anxiety disorder and is therefore largely prescribed by neurologists, psychiatrists and, of course, general practitioners. Apart from the most common side effects such as—among others—drowsiness, increased appetite, and headache, also depressive mood disorders must be kept in mind as side effects of pregabalin and various other antiepileptic drugs. An FDA warning concerning self-harm in patients taking antiepileptic drugs was released in 2008 [1], yet a relevant risk for pregabalin is postulated to be less than 1% [2] (low-risk group of antiepileptic compounds concerning mood disorders). An observational study [2] did not find a significant increase in self-harm within the group of those low-risk compounds; however, a review of the literature indicated that suicidal ideations under pregabalin have recently been reported in one case [3] under a daily dose of 600 mg pregabalin. The following case is startling inasmuch as the patient did not only experience suicidal ideations but did attempt suicide while being prescribed pregabalin.

## Case presentation

The patient we present is a 20-year-old male admitted to our psychiatric ward in February who reported depressed mood, loss of motivation, hopelessness, anxiety, sleep disturbances, and suicidal thoughts as major depressive symptoms.

Recent brain imaging, as well as routine laboratory analysis upon admission, was unremarkable.

It was the patient’s first episode of a psychiatric disorder. It originally started with somatic symptoms and their treatment. He suffered from an orofacial dyskinesia of unknown origin, muscular atrophy of the right forearm, and an alar scapula on the left, currently diagnosed as multifocal motoric neuropathia. The orofacial dyskinesia was first treated symptomatically with tiapride starting in July the year before admission, which he tolerated well. Later, the movements were thought to be complex-focal seizures, the medication was switched to pregabalin, 150 mg daily within 2 weeks during his stay in an external clinic in October. The patient reported no further co-medication at that time.

Starting with the first prescription of pregabalin in October, the patient felt increasingly depressed (visual analogue scale 4/10–5/10) and demotivated. Suicidal thoughts first appeared in November. These continued to worsen and led to a suicide attempt in December of the year before admission; he tried to poison himself to death using high doses of ibuprofen and aspirin after consuming alcohol. He could not name any triggering factors and described the incident as a completely unexpected and irrational act. He was seen by his general practitioner and taken care of by his family. He did not receive further inpatient treatment then but was prescribed antidepressants for the emerging mood disorder starting with the sedating mirtazapine 15 mg at night in order to prevent further harmful acts on impulse. Later, the serotonergic drug citalopram was added in order to address the lack of motivation, starting with 10 mg and rising to 20 mg in the morning. Tiapride was also restarted for controlling the dyskinesia.

After the patient retrospectively clearly correlated the start of pregabalin therapy with the onset of his depressive symptoms during exploration on our ward, we immediately discontinued the drug. Consequently, he reported a rapid decline in depressive symptoms, his mood remained stable, and he was discharged. Until then, pregabalin was not believed to trigger the patient’s symptoms, and his somatic problems were the focus of investigation and treatment. Thus, there are unfortunately no objective follow-up scales available but only symptom descriptions. His alcohol intake preceding the suicide attempt may be considered a confounding factor. However, casual drinking before taking the drug never caused suicidal thoughts prior to this episode. The depressive symptoms themselves began only when the drug was started; thus, alcohol might have had an aggravating effect.

## Conclusions

For the clinician, the case illustrates that not only suicidal ideations—a known but rare adverse effect of pregabalin—but also suicidal actions under pregabalin therapy have to be kept in mind. The issue that alcohol can reduce one’s inhibitions to actually carry out suicidal ideations should be separately addressed in the dialogue with the patient and the combination with pregabalin should strictly be advised against.

For theoretical considerations, it is quite surprising that a compound relieving a patient from his anxious distress sometimes does not improve mood and activity but, on the contrary, causes severe depression and self-harm tendencies. Taking into account that certain antiepileptic drugs have been shown to be protective for patients with bipolar disorder concerning suicidality [4], but hazardous for patients with monopolar depression, for example [5], there may be critical patient variables also for pregabalin which remain to be elucidated.

In the current case, pregabalin has been increased to the daily dose of 150 mg within 2 weeks. In order to prevent more common side effects of the compound such as drowsiness or headache, a low starting dose which is then gently increased often turns out to be helpful. Maybe the initial dosing regimen also plays a role with the depressive side effects.

Concerning the pharmacodynamic side of the mood effects of antiepileptic drugs, serotonergic pathways are discussed [6]. Regarding analgesic efficacy of pregabalin, it has been shown to depend on the descending serotonergic system with spinal 5HT3 receptors [7], so serotonergic effects may also play a role for pregabalin-induced mood alterations.

## Consent

We thank the patient for providing his written consent to publish this case report.
